# Evaluating Proton Dose and Associated Range Uncertainty Using Daily Cone-Beam CT

**DOI:** 10.3389/fonc.2022.830981

**Published:** 2022-04-05

**Authors:** Heng Li, William T. Hrinivich, Hao Chen, Khadija Sheikh, Meng Wei Ho, Rachel Ger, Dezhi Liu, Russell Kenneth Hales, Khinh Ranh Voong, Aditya Halthore, Curtiland Deville

**Affiliations:** Radiation Oncology and Molecular Radiation Sciences, Johns Hopkins University School of Medicine, Baltimore, MD, United States

**Keywords:** dose calculation, CBCT, radiation measurement, radiation therapy, proton radiation therapy (PBT)

## Abstract

**Purpose:**

This study aimed to quantitatively evaluate the range uncertainties that arise from daily cone-beam CT (CBCT) images for proton dose calculation compared to CT using a measurement-based technique.

**Methods:**

For head and thorax phantoms, wedge-shaped intensity-modulated proton therapy (IMPT) treatment plans were created such that the gradient of the wedge intersected and was measured with a 2D ion chamber array. The measured 2D dose distributions were compared with 2D dose planes extracted from the dose distributions using the IMPT plan calculated on CT and CBCT. Treatment plans of a thymoma cancer patient treated with breath-hold (BH) IMPT were recalculated on 28 CBCTs and 9 CTs, and the resulting dose distributions were compared.

**Results:**

The range uncertainties for the head phantom were determined to be 1.2% with CBCT, compared to 0.5% for CT, whereas the range uncertainties for the thorax phantom were 2.1% with CBCT, compared to 0.8% for CT. The doses calculated on CBCT and CT were similar with similar anatomy changes. For the thymoma patient, the primary source of anatomy change was the BH uncertainty, which could be up to 8 mm in the superior–inferior (SI) direction.

**Conclusion:**

We developed a measurement-based range uncertainty evaluation method with high sensitivity and used it to validate the accuracy of CBCT-based range and dose calculation. Our study demonstrated that the CBCT-based dose calculation could be used for daily dose validation in selected proton patients.

## Introduction

The use of daily cone-beam CT (CBCT) guidance has improved patient setup accuracy and precision in various radiotherapy delivery modalities, including proton therapy (1, 2). Since the proton range, and hence the proton dose distribution, is sensitive toward any density change along the beam path, CBCT can provide an additional benefit of verifying proton range and dose distribution, complementing the geometrical verification of the patient anatomy daily (3–5). However, the Hounsfield unit (HU) accuracy and image quality of the CBCT images are inferior to those of regular CT and often considered inadequate for proton dose calculation without correction. Methods to correct the HU of CBCT and generate synthetic CT from CBCT images using regular CT images as *a priori* information have been extensively studied (6–8). However, problems arise when there is a difference between CBCT and regular CT. The CBCT data could be skewed and biased toward the regular CT, masking the very information we seek from CBCT. For example, Veiga et al. proposed to use deformable image registration (DIR) to create virtual CT (vCT) from CBCT and planning CT (pCT) (8–10). However, an additional correction step, which has an unknown impact on the final result, was necessary because the DIR process could artificially change the patient’s anatomy as observed on CBCT (8). In general, when *a priori* information such as pCT was used, the bias it introduced and its impact on the range and dose distribution would compound with the actual anatomy change of the patient and could not be adequately quantified. On the other hand, previous studies have shown that using CBCT HU to density table (HU-D table) could produce mass density and dose calculation results with accuracy on the order of 1% for intensity-modulated radiation (photon) therapy (IMRT) treatments, even with inferior HU accuracy compared to CT (11). However, for proton radiotherapy, the proton range uncertainty arising from CBCT images with HU to density conversion instead of HU correction, and its impact on dose calculation, has not been quantitatively studied (12).

This study proposes using a measurement-based technique to quantify the proton range uncertainties arising from CBCT HU to density conversion, as compared to helical CT. We will also demonstrate the feasibility of CBCT-based daily dose validation without HU correction using a patient case.

## Materials and Methods

### Imaging Systems and Proton Dose Calculation

The RayStation treatment planning system (versions 9A/10A, RaySearch Laboratories, Stockholm, Sweden) was used for this study. Proton dose engines in RayStation require a complete description of the material composition, including mass density, mass fraction of atomic elements, and mean ionization energy for each voxel of the patient. With the use of CT images as input, this was implemented by converting HU to mass density with the HU-D table. Then, the mass fraction of atomic elements and mean ionization energy of the voxel were determined from a number of well-established core materials through the interpolation of mass density (13).

A stoichiometric calibration method (14, 15) was used to establish the HU-D table for the CT simulator used in this study (Siemens SOMATOM Definition Edge plus, Siemens Healthcare, Forchheim, Germany). Separately, a patient group-based method (11) was used to create the HU-D table for the CBCT system used in this study (integrated into the Hitachi Probeat CR proton delivery system, Hitachi, Ltd., Tokyo, Japan). CT and CBCT image datasets of brain, head and neck, and thorax patients were used to establish the HU-D relationship on the CBCT images for typical materials such as air, brain, bone, or lung. The HU-D tables for CT and CBCT were then used for proton dose calculation on images acquired with respective modalities.

### Phantom Imaging

**Figure 1:**
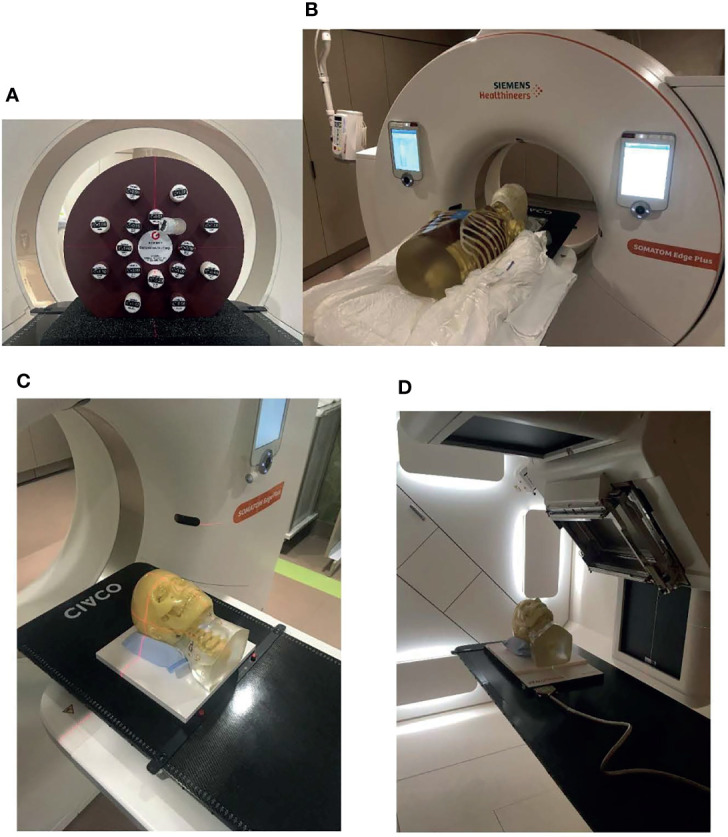
Phantoms that were used in the study. **(A)** CT density phantom, **(B)** head and thorax phantoms put together on the simulator, **(C)** the head phantom on top of a solid water slab at the simulator, and **(D)** the head phantom on top of a solid water slab and a 2D ion chamber array at the treatment position ready for cone-beam CT (CBCT).

A CT electron density phantom with inserts of known density (Gammex Inc., Middleton, WI, USA) and two anthropomorphic phantoms (Radiological Support Devices Inc., Long Beach, CA, USA) (one head phantom and one thorax phantom) were used in this study. **Figure 1A** shows the CT electron density phantom, **Figure 1B** shows the head and thorax phantoms put together on the simulator, **Figure 1C** shows the head phantom on top of a solid water slab at the simulator, and **Figure 1D** shows the head phantom on top of a solid water slab and a 2D ion chamber array (Octavius, PTW, Freiburg, Germany) at the treatment position ready for CBCT.

### Phantom Treatment Planning

**Figure 2:**
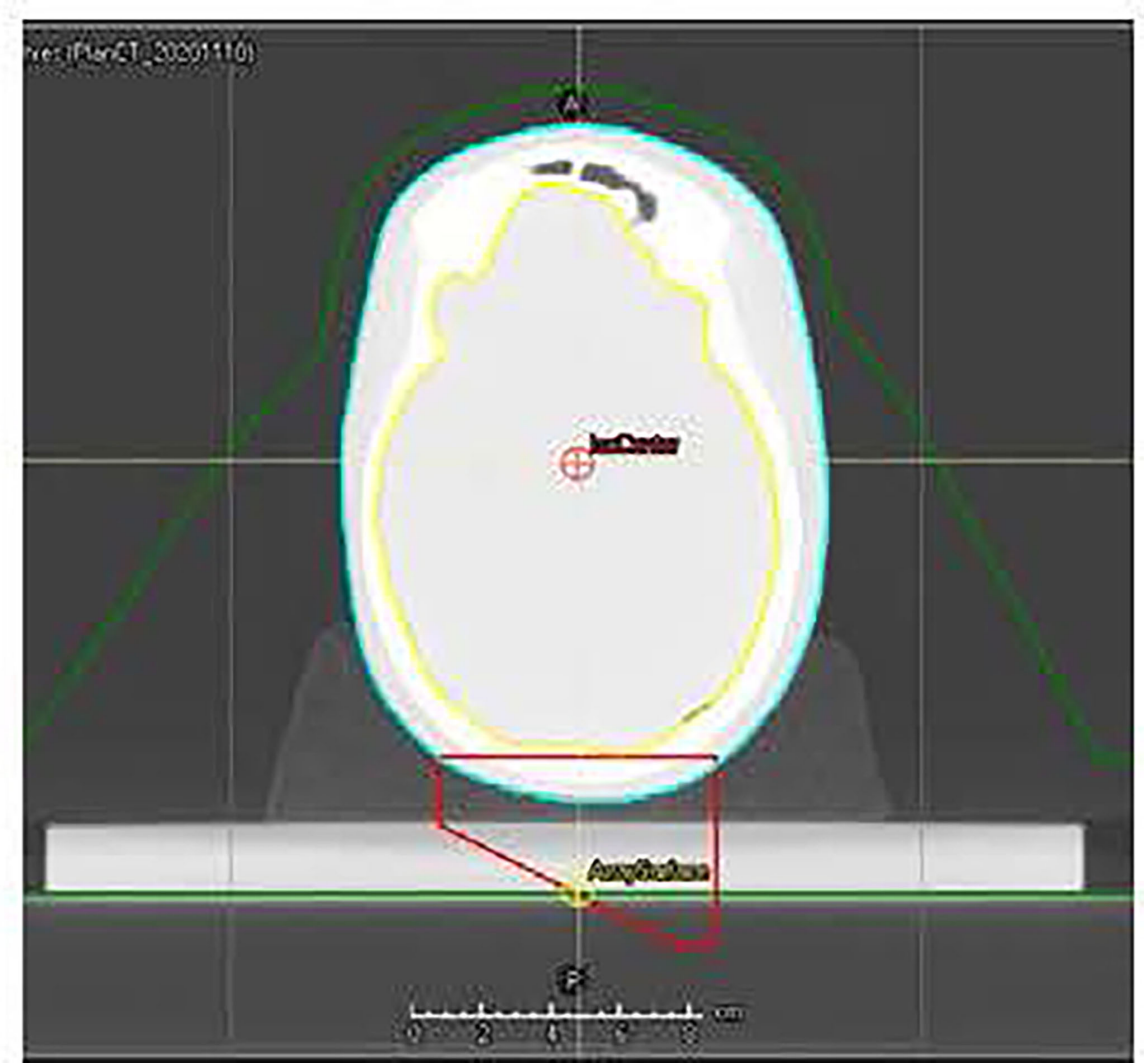
CT image of the head phantom with a wedge-shaped target.

The pCT images were sent to RayStation. A solid water “Base” support was inserted posterior to the phantom, as it would be placed on top of the detector array during delivery. A wedge-shaped clinical target volume (CTV) was added posterior to the head such that the gradient of the wedge intersected the solid water slabs, as shown in **Figure 2**. This target design was chosen so that range error could be estimated from the gradient region of the wedge structure as measured using the detector array. For example, if the gradient edge was measured further laterally than computed by the TPS, this would indicate a measurement range deeper than the TPS predicted. A single anterior–posterior (AP) beam was optimized to deliver a homogeneous dose of 200 cGy (relative biological effectiveness (RBE)) to the wedge target, with robust optimization parameters of 0-mm setup uncertainties and 3.5% range uncertainties. The final dose was computed using a Monte Carlo proton dose engine with a 1-mm isotropic dose grid and exported in RT DICOM format to the Oncology Information System (OIS, Mosaiq, Elekta, Crawley, UK).

### Phantom Setup and Cone-Beam CT Imaging

The head phantom was then set up to the “treatment” position, as shown in **Figure 1C** with laser, followed by shifts guided by a 3D–3D rigid registration between the CBCT and the plan CT. The CBCT images and the rigid registration were sent to OIS and RT PACS (Evercore DICOM RT archive, TeraMedica, Milwaukee, WI, USA).

### Phantom Measurement

The treatment plan was then delivered using the proton treatment delivery system. A 2D plane of the delivered dose was measured using the 2D ion chamber array. A single 1-cm slab of solid water was placed between the phantom headrest and the detector array to ensure that the gradient region of the wedge intersected the detector array, as shown in **Figure 1C**.

### Hounsfield Unit Difference Between CT and Cone-Beam CT Images

The HUs of the CT and CBCT images for CT density inserts and patient images were compared. The difference of HU for the same material on the CT and CBCT images leads to different HU-D calibrations for the two different modalities.

### Phantom Cone-Beam CT-Based Dose Calculation

The acquired CBCT images and the rigid registration were sent to the TPS from PACS. The proton plan was recalculated on CBCT with the isocenter placed using the registration.

### Phantom Quantification of Range Uncertainties From CT and Cone-Beam CT

The proton dose distribution calculated on CT and CBCT was exported from the TPS. The measured 2D dose planes with the CT and CBCT doses were compared using Matlab (MathWorks, Inc., Natick, MA, USA). The measurement plane was determined on CT and CBCT, respectively. The corresponding dose planes on CT and CBCT were extracted and compared with the 2D measurement by calculating the gamma indexes with criteria of 3 mm/3%. Because of the range uncertainties associated with the HU-D-proton stopping power conversion process, the measured dose plane may be shifted only on the range direction, assuming no geometrical uncertainties. Therefore, the range uncertainties between the calculated dose and the measurement could be quantified by the difference between the calculated measurement depth and the actual measurement depth. The actual measurement depth could be determined by comparing various dose planes extracted from the 3D dose calculated on CT/CBCT with the measured 2D dose plane.

Determination of the range uncertainties from CT and CBCT was repeated for head and thorax phantoms.

### Workflow of Cone-Beam CT-Based Dose Verification

**Figure 3:**
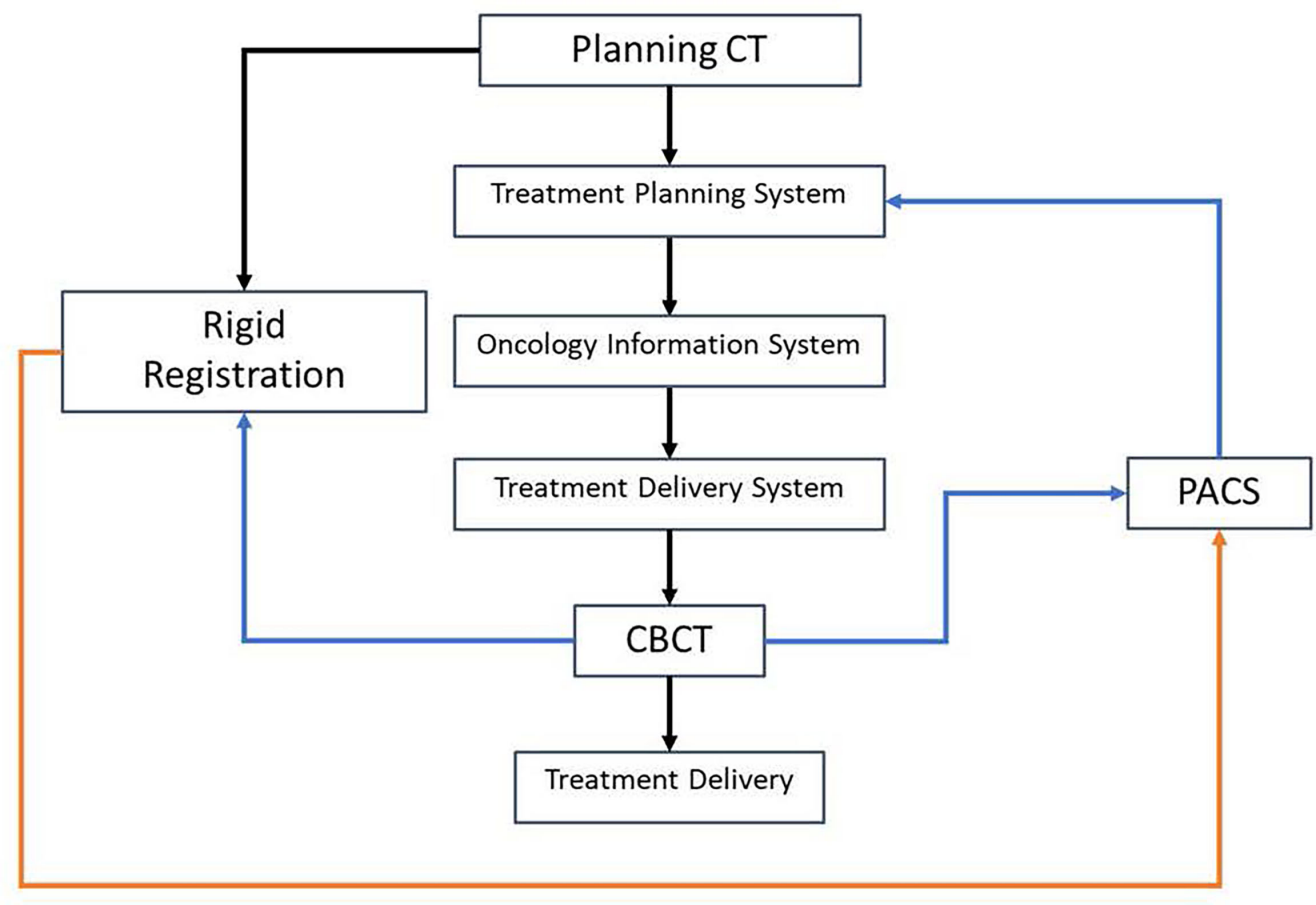
Workflow of using cone-beam CT (CBCT) for patient dose verification. The black lines represent the data transfer of planning CT and the treatment plan, the blue line represents the data transfer of CBCT, and the orange line represents the data transfer of rigid registration.

**Figure 3** shows the workflow of using CBCT for patient dose verification. Black lines represent the data transfer of pCT and the treatment plan, the blue line represents the data transfer of CBCT, and the orange line represents the data transfer of rigid registration. The workflow is similar to the phantom validation workflow described above, and a large part of the workflow could be automated through scripting.

### Patient Case Study

A patient with thymoma treated with active breathing control (ABC) (16), which is a breath-hold (BH) technique, and daily CBCT-guided intensity-modulated proton therapy (IMPT) was retrospectively studied. Three consecutive BH CT scans were acquired during patient simulation for treatment planning. Additionally, the patient acquired two additional sets of repeated CTs throughout the treatment, each consisting of three consecutive BH scans. The patient was given a total dose to CTV of 5,400-cGy RBE in 27 fractions. The patient went through CBCT imaging the first day on the treatment machine without actual treatment delivery, followed by daily CBCT-guided proton therapy, resulting in an additional set of CBCT images. For daily CBCT acquired with BH, the patient held their breath for ~25 s for each BH, and each full scan of CBCT acquisition required ~3–4 BH. In summary, there were 9 CTs and 28 CBCTs available for the patient.

Two treatment plans were created for the patient with robustly optimized IMPT using a spot scanning technique (17, 18). The first plan was created with only one of the planning BH CTs (19), whereas the second plan used all three planning BH CTs with multiple-CT optimization (20, 21). With the IMPT technique, all surrounding organs at risk (OARs), including the lung and the heart, received doses well below clinical tolerance in both plans. The main challenge was maintaining target coverage with the intra-fractional and inter-fractional motions of the patient. The proton plans were recalculated on all 9 CT and 28 CBCT scans to monitor and evaluate the impact of the motion. The target coverage along with heart and lung doses on each scan was evaluated.

## Results

### Phantom Study

**Figure 4:**
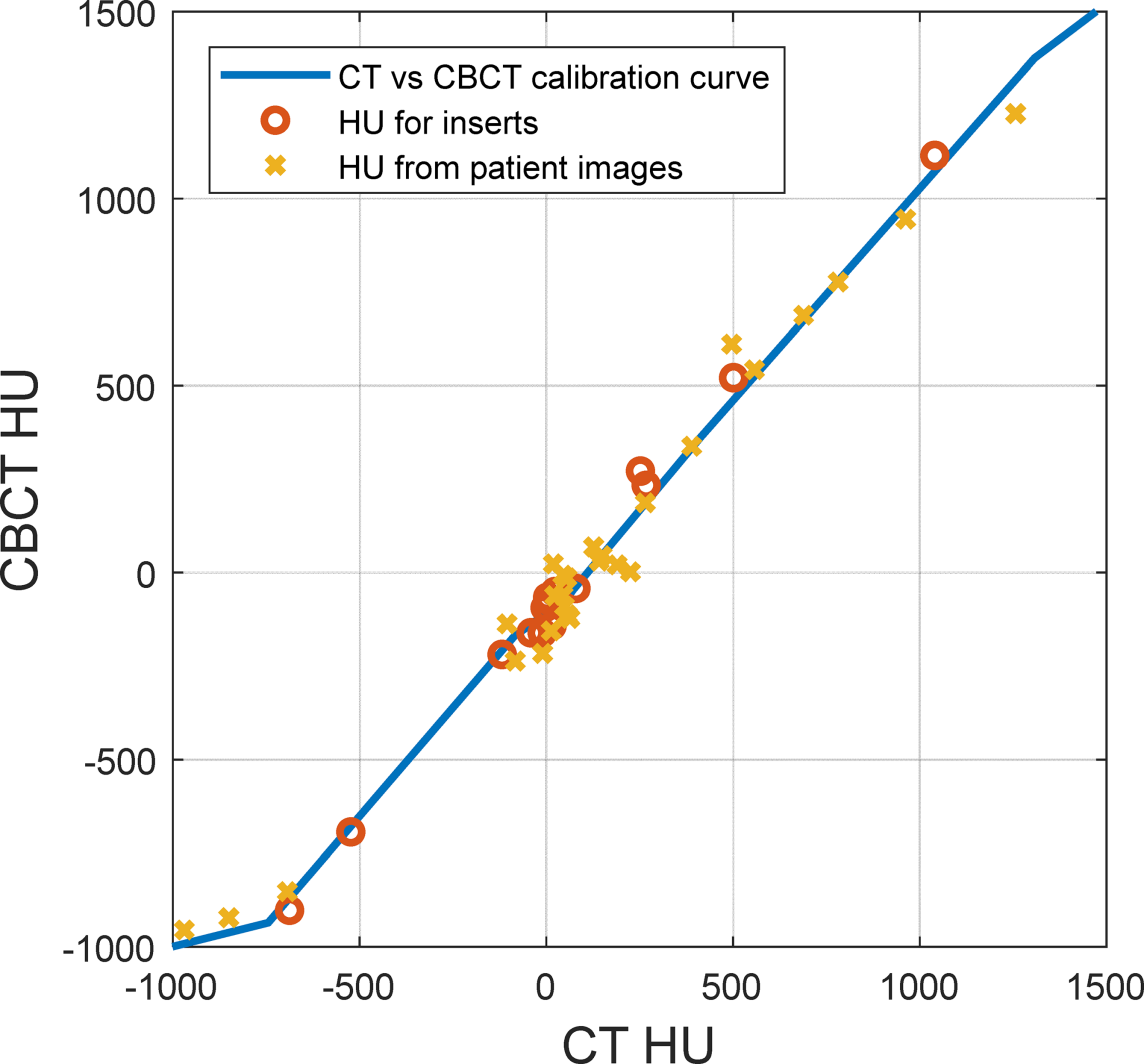
Hounsfield unit (HU) comparison between CT and cone-beam CT (CBCT) for the same materials. The solid line represents the HU-D calibration curve, the circles represent inserts on the CT density phantom with known densities, and the crosses represent volumes of interest with nearly homogenous density on patient images.

**Figure 4** shows the comparison between HU from CT and CBCT for the same materials. The figure shows the HUs for inserts in the CT electron density phantom with known densities, extracted from CT images (x-axis) and CBCT images (y-axis). It also shows the HUs of volumes of interest (VOI) with nearly homogenous densities on the reference CT and the daily CBCT images (11), along with the HU-D curves for CT and CBCT modalities. There is a substantial difference between HUs from CT and CBCT for lung and soft tissue materials.

**Figure 5:**
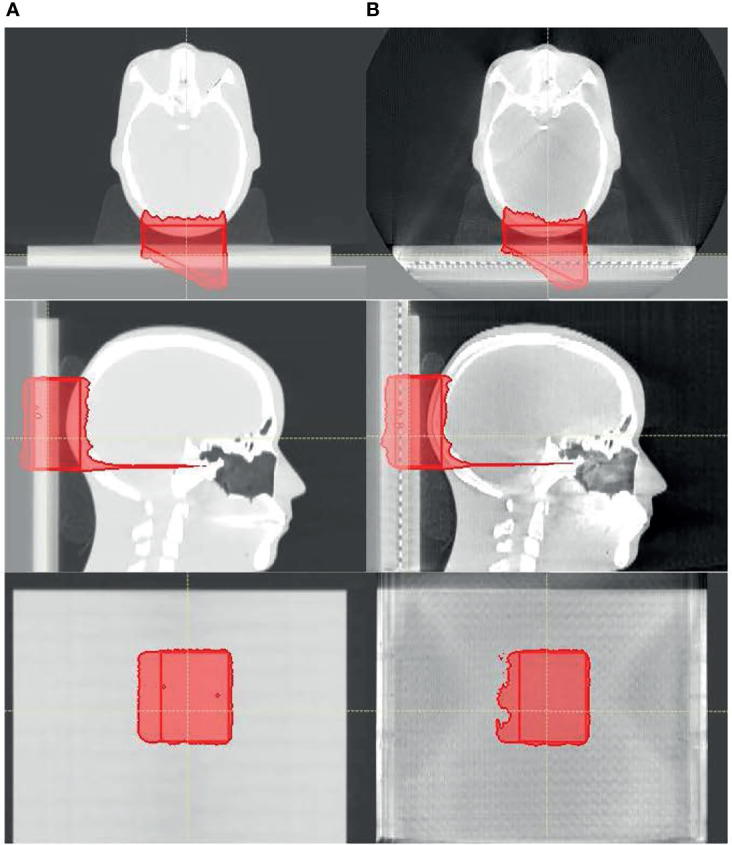
The proton dose distribution calculated on **(A)** planning CT and **(B)** cone-beam CT (CBCT) for the head phantom. Only the prescription dose of 200 cGy is shown in the red color wash, and the red contour represents the target.

**Figure 5** shows the proton dose distribution calculated on pCT (**Figure 5A**) and CBCT (**Figure 5B**) for the head phantom. The prescription dose of 200-cGy RBE is shown in the red color wash, and the red contour represents the target. As shown in the figures, since zero setup uncertainties were used in treatment planning, the prescription dose highly conformed to the target contour on lateral directions of the beam. Since 3.5% range uncertainties were used, there were margins on the proximal and distal directions of the beam between the prescription dose and the target. The 2D detector array could be easily identified from the CBCT. Since the entire base was overridden as solid water (shown in the figures as gray blocks), the material composition difference between CT and CBCT was not of concern.

**Figure 6:**
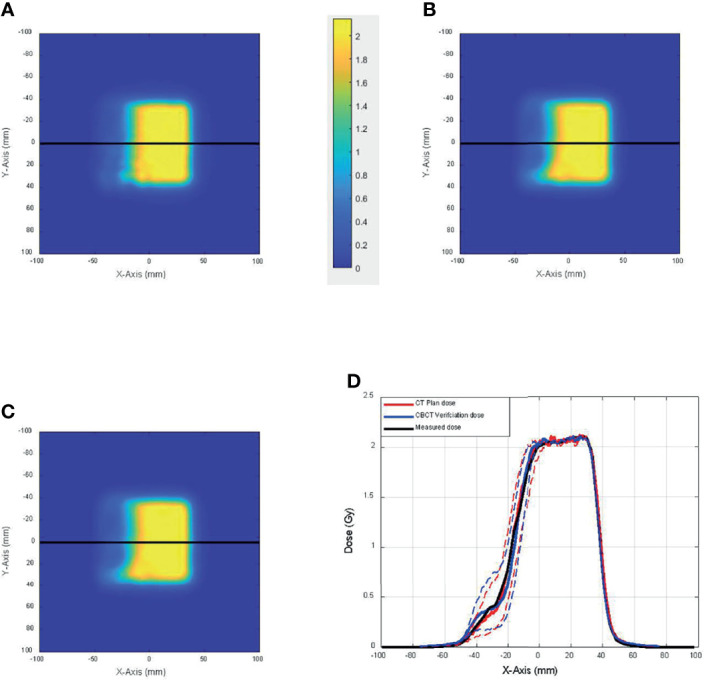
The comparison among 2D planes extracted from the 3D dose calculated on **(A)** CT and **(B)** cone-beam CT (CBCT) that best match **(C)** the 2D plane measurement. **(D)** Dose profile of panels A–C (solid lines) and dose profiles from CT and CBCT dose planes that were 3 mm proximal and distal toward the best-matched dose planes (dashed lines).

As described in the *Materials and Methods* section, 2D dose planes from the 3D dose on CT and CBCT were extracted in 1-mm spacing and compared with the measured 2D plane dose. **Figure 6** shows the comparison among 2D planes extracted from the 3D dose calculated on CT (**Figure 6A**) and CBCT (**Figure 6B**) that best matches the 2D plane measurement (**Figure 6C**). **Figure 6D** shows the dose profile of **Figures 6A–C**, which were again the 2D array measurement along with the best-matched dose planes from dose calculation using CT and CBCT (solid lines). Also shown in the figure, in dashed lines, are the dose profiles from CT and CBCT dose planes that were 3 mm proximal and distal toward the best-matched dose planes. This figure demonstrates the geometric accuracy of the comparison and the sensitivity of the test toward range uncertainties.

**Figure 7:**
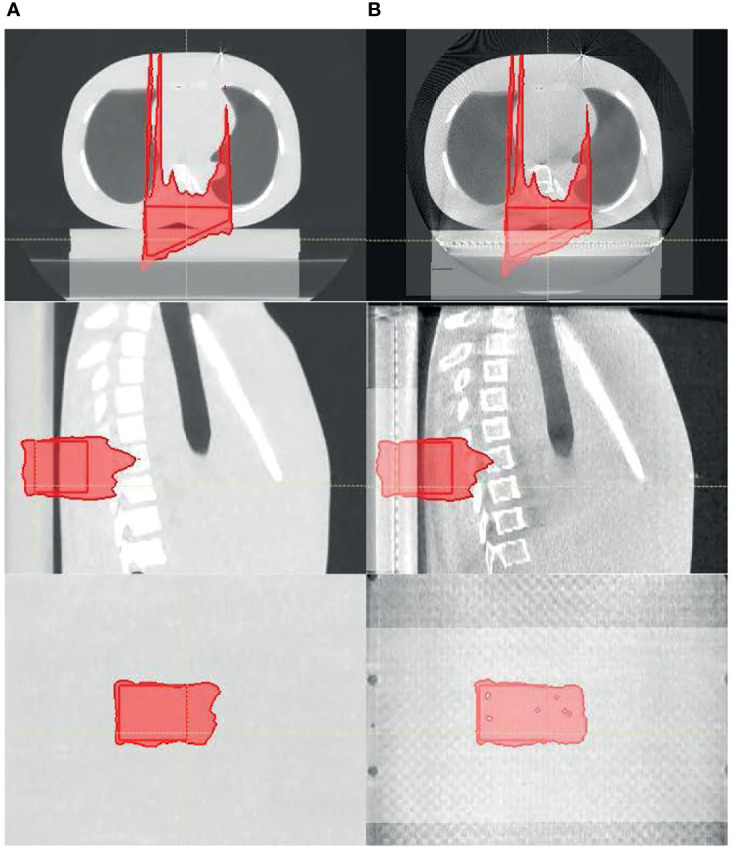
The proton dose distribution calculated on **(A)** planning CT and **(B)** cone-beam CT (CBCT) for the thorax phantom.

**Figure 8:**
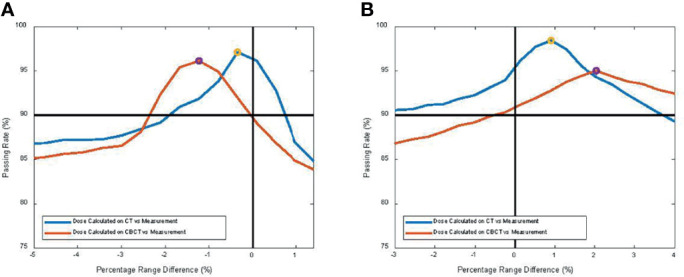
2D gamma passing rate with criterion of 3 mm/3% as a function of percentage range error, comparing the 2D measurement and 2D dose planes from CT or cone-beam CT (CBCT) dose for **(A)** the head phantom and **(B)** the thorax phantom, respectively.

**Figure 7** shows the proton dose distribution calculated on pCT (**Figure 7A**) and CBCT (**Figure 7B**) for the thorax phantom. **Figure 8** shows the 2D gamma passing rate with a criterion of 3 mm/3% as a function of percentage range error, comparing the 2D measurement and 2D dose planes from CT or CBCT dose, for the head phantom (**Figure 8A**) and the thorax phantom (**Figure 8B**), respectively. The range error from CT was 0.5% and 0.8%, whereas the range error from CBCT was 1.2% and 2.1% for the head and thorax phantoms, respectively.

### Patient Study

**Figure 9:**
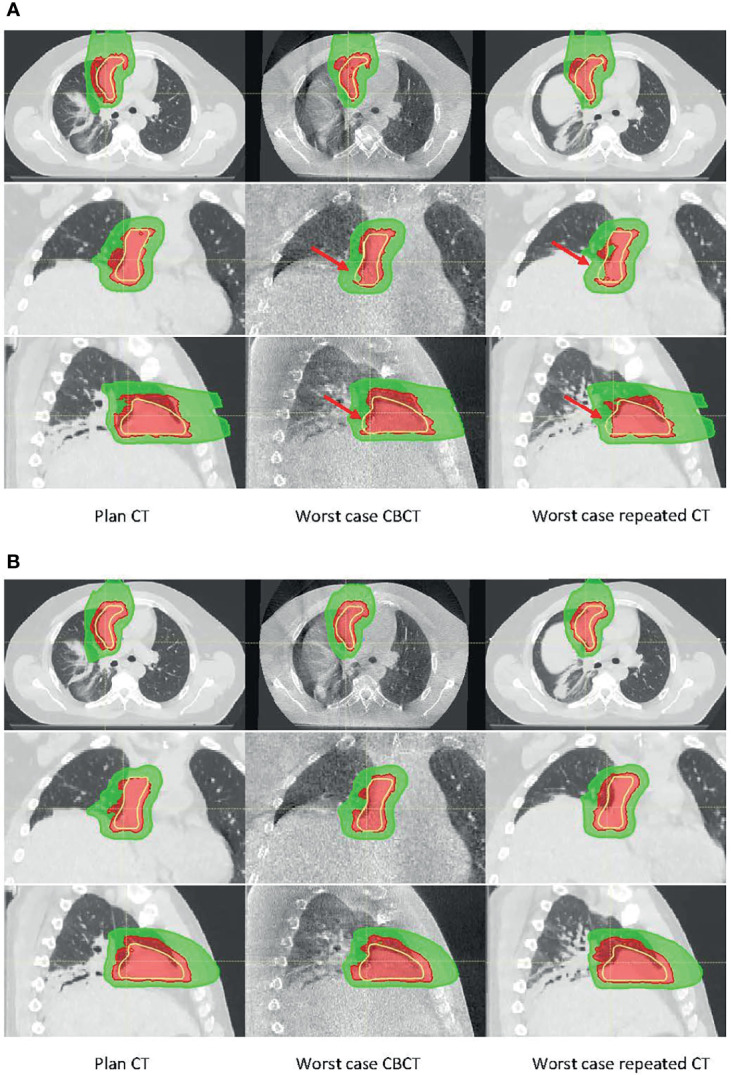
Dose distribution calculated on planning CT (left), worst-case cone-beam CT (CBCT) (middle), and worst-case repeated CT (right) for **(A)** the single breath-hold BH CT plan and **(B)** multiple BH CT plan. There were ~8-mm superior–inferior (SI) BH uncertainties between the planning CT and the worst-case CBCT or CT. Red arrows in panel A point to the area where target coverage was reduced.

Two treatment plans were based on one ABC BH CT and on multiple (three) ABC BH scans. The first column of **Figures 9A, B** shows the dose distribution on the single pCT and multiple BH CTs, respectively. In the figures, only the dose cloud of the prescription dose (5,400-cGy RBE, red) and 50% (2,700-cGy RBE, green) are shown along with the CTV (yellow contour). The plan was recalculated on all 9 CTs and 28 CBCTs. The middle and right columns of **Figures 9A, B** show the dose distributions on CBCT and CT with the worst CTV coverage, respectively. As observed from CBCT and CT, the BH uncertainty was the primary source of anatomy change in the patient. The BH level could differ up to 8 mm in the superior–inferior (SI) direction. Red arrows in **Figure 9A** point to areas where target coverage was reduced on the daily CBCT or repeated CT. The treatment plan based on a single BH CT could not account for the anatomy change in the worst-case CBCT and CT; both result in loss of tumor dose coverage, whereas the dose coverage was maintained for the multiple BH CT plan. These results suggest that CBCT could provide similar information as repeated CT to support the clinical decision on adaptation.

**Figure 10:**
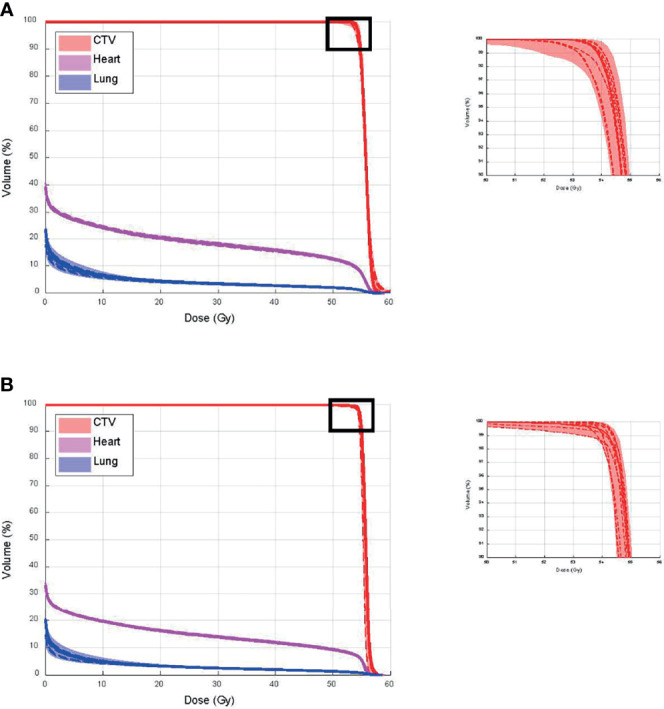
Clinical target volume (CTV), and heart and lung dose–volume histograms (DVHs) for **(A)** single CT and **(B)** multiple CT plans, calculated on all CTs and cone-beam CTs (CBCTs), respectively.

**Figure 10** shows CTV, and heart and lung dose–volume histograms (DVHs) for the single CT (**Figure 10A**) and multiple CT (**Figure 10B**) plans, calculated on all CTs and CBCTs, respectively. The solid line represents DVHs on pCT, the dashed lines represent DVHs on all CTs, and the bands represent DVHs on all CBCTs. The figure shows that V100% from the single CT plan dropped to 95.0% and 94.5%, in the worst case on CTs and CBCTs, respectively. The V100% for the multiple CT plan dropped to 98.4% for both CTs and CBCTs. Both plans’ heart and lung doses remained excellent on all CTs and CBCTs.

## Discussion

In this study, we quantified the proton range uncertainties and their impact on dose calculation arising from CBCT images with HU to density calibration for the first time. The range uncertainties for the head phantom were determined to be 1.2% with CBCT, compared to 0.5% for CT, whereas the range uncertainties for the thorax phantom were 2.1% with CBCT, compared to 0.8% for CT. The profile comparison in **Figure 6D** and the gamma index comparison in **Figure 8** both showed excellent resolution of the measurement-based technique, in terms of both general geometric accuracy and the determination of range uncertainties based on dose gradient. These results are comparable with synthetic CT techniques for head (22) and thorax (8) studies.

**Figure 11:**
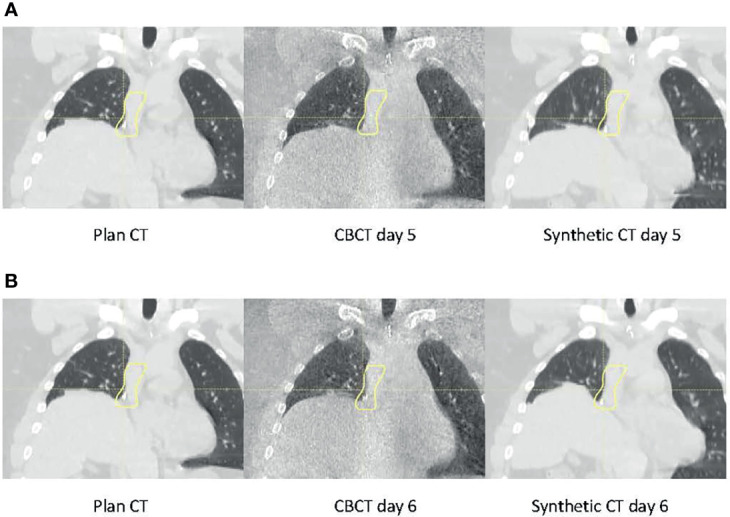
Planning CT (left), daily cone-beam CT (CBCT (middle), and synthetic CT (right) for the same patient on **(A)** day 5 and **(B)** day 6.

One notable difference between the current and previous studies is that measurement was used as the baseline for our study. Much of the literature investigating CBCT-based proton dose calculation converted CBCT HU to CT HU and then used CT HU for proton dose calculation (3, 23–27). However, as mentioned above and demonstrated in this study, using CT as the baseline would introduce the inherent range uncertainties associated with the CT HU-D-stopping power conversion, which could potentially be removed by direct CBCT HU-D-stopping power conversion. Many HU correction techniques also use CT as *a priori* information that, as previously discussed, could introduce bias and geometrical uncertainties, which could compound with the actual patient anatomy change. Since the uncertainties could not be adequately quantified, one of the major benefits of CBCT images (i.e., its excellent geometrical accuracy) would be negated. **Figure 11** demonstrates an example of geometric uncertainties from the DIR-based HU correction techniques. In the figure, the planning ABC CT (left) used *a priori* information to correct the CBCT images (center) using deformation registration to create the synthetic CT images (right) on two consecutive days at day 5 (**Figure 11A**) and day 6 (**Figure 11B**), respectively (8). While the HU accuracy was generally improved on the synthetic CT compared to CBCT, the diaphragm was displaced in the synthetic CT on day 6. Since pCT is not the ground truth for daily patient data, the magnitude of the geometrical distortion in the synthetic CT could not be adequately quantified. This observation is consistent with previous publications (8–10). Advanced techniques for CBCT HU to density conversion without distorting the patient geometry could further improve the accuracy of the CBCT-based dose calculation. For example, Spadea et al. and Thummerer et al. (22, 28) proposed a deep convolutional neural network based on CBCT HU to CT HU mapping to create synthetic CT without introducing geometry distortion.

We compared the dose calculated on CBCTs with CTs for an ABC BH patient as a feasibility study. The results showed that the CBCT calculated dose was consistent with CT calculated dose when there were similar BH-induced uncertainties. These results suggest that with a simple workflow, CBCT-based dose validation could be unbiased with excellent geometrical accuracy, and acceptable accuracy range determination, and may be an alternative to synthetic CT-based dose validation for selected proton patients.

In addition to the inferior image quality, CBCT also has other inherent problems that could limit the use of CBCT directly for dose calculation. For example, CBCT image quality could deteriorate with artifacts from metal or motion. One of the other major limitations of CBCT is the limited field of view (FOV). For accurate proton dose calculation, the entire beam path that transverses the patient and any supporting devices need to be included in the FOV of the images, which may not be feasible for many patients with pelvic targets. In addition, the HU accuracy of CBCT images is known to deteriorate with patient size. Therefore, we limited our investigation to head and thorax phantoms in this study. We do not expect to use CBCT directly for dose calculation in large patients where the FOV could not cover the entire patient.

## Conclusion

We developed a measurement-based range uncertainty evaluation method with high sensitivity and used it to validate the accuracy of CBCT-based range and dose calculation. Our study demonstrated that the CBCT-based dose calculation could be used for daily dose validation in selected proton patients.

## Data Availability Statement

The raw data supporting the conclusions of this article will be made available by the authors, without undue reservation.

## Ethics Statement

The retrospective study was based on patient data with Johns Hopkins Medicine IRB approval.

## Author Contributions

HL, WH, and HC conceptualized the design and experiments. HL and WH drafted the manuscript. HC, WH, and KS contributed to acquiring, analyzing, and interpreting the data. MH and DL contributed to the coding of the analysis. RH, KV, and AH provided recommendations on the clinical perspective of the study. RG and CD reviewed and edited the manuscript. All authors read and approved the final manuscript.

## Conflict of Interest

The authors declare that the research was conducted in the absence of any commercial or financial relationships that could be construed as a potential conflict of interest.

## Publisher’s Note

All claims expressed in this article are solely those of the authors and do not necessarily represent those of their affiliated organizations, or those of the publisher, the editors and the reviewers. Any product that may be evaluated in this article, or claim that may be made by its manufacturer, is not guaranteed or endorsed by the publisher.

## References

[1] Hua C Yao W Kidani T Tomida K Ozawa S Nishimura T. A Robotic C-Arm Cone Beam CT System for Image-Guided Proton Therapy: Design and Performance. Br J Radiol (2017) 90:20170266. doi: 10.1259/bjr.20170266 28830239 PMC5963391

[2] MacFarlane MJ Jiang K Mundis M Nichols E Gopal A Chen S. Comparison of the Dosimetric Accuracy of Proton Breast Treatment Plans Delivered With SGRT and CBCT Setups. J Appl Clin Med Phys (2021) 22:153–8. doi: 10.1002/acm2.13357 PMC8425866 34288378

[3] Park YK Sharp GC Phillips J Winey BA. Proton Dose Calculation on Scatter-Corrected CBCT Image: Feasibility Study for Adaptive Proton Therapy. Med Phys (2015) 42:4449–59. doi: 10.1118/1.4923179 PMC4517932 26233175

[4] Nesteruk KP Bobić M Lalonde A Winey BA Lomax AJ Paganetti H. CT-on-Rails Versus in-Room CBCT for Online Daily Adaptive Proton Therapy of Head-and-Neck Cancers. Cancers (2021) 13:5991. doi: 10.3390/cancers13235991 34885100 PMC8656713

[5] Paganetti H Botas P Sharp GC Winey B. Adaptive Proton Therapy. Phys Med Biol (2021) 66:22TR01. doi: 10.1088/1361-6560/ac344f PMC8628198 34710858

[6] Landry G Dedes G Zöllner C Handrack J Janssens G De Xivry JO. Phantom Based Evaluation of CT to CBCT Image Registration for Proton Therapy Dose Recalculation. Phys Med Biol (2014) 60:595. doi: 10.1088/0031-9155/60/2/595 25548912

[7] Landry G Nijhuis R Dedes G Handrack J Thieke C Janssens G. Investigating CT to CBCT Image Registration for Head and Neck Proton Therapy as a Tool for Daily Dose Recalculation. Med Phys (2015) 42:1354–66. doi: 10.1118/1.4908223 25735290

[8] Veiga C Janssens G Teng C-L Baudier T Hotoiu L Mcclelland JR. First Clinical Investigation of Cone Beam Computed Tomography and Deformable Registration for Adaptive Proton Therapy for Lung Cancer. Int J Radiat Oncol Biol Phys (2016) 95:549–59. doi: 10.1016/j.ijrobp.2016.01.055 27084664

[9] Veiga C Janssens G Baudier T Hotoiu L Brousmiche S Mcclelland J. A Comprehensive Evaluation of the Accuracy of CBCT and Deformable Registration Based Dose Calculation in Lung Proton Therapy. Biomed Phys Eng Express (2017) 3:015003. doi: 10.1088/2057-1976/3/1/015003

[10] Veiga C Alshaikhi J Amos R Lourenço AM Modat M Ourselin S. Cone-Beam Computed Tomography and Deformable Registration-Based “Dose of the Day” Calculations for Adaptive Proton Therapy. Int J Particle Ther (2015) 2:404–14. doi: 10.14338/IJPT-14-00024.1

[11] Richter A Hu Q Steglich D Baier K Wilbert J Guckenberger M. Investigation of the Usability of Conebeam CT Data Sets for Dose Calculation. Radiat Oncol (2008) 3:1–13. doi: 10.1186/1748-717X-3-42 19087250 PMC2648965

[12] Giacometti V Hounsell AH McGarry CK. A Review of Dose Calculation Approaches With Cone Beam CT in Photon and Proton Therapy. Physica Med (2020) 76:243–76. doi: 10.1016/j.ejmp.2020.06.017 32736286

[13] Raysearch Laboratories. RSL-D-RS-9A-REF-EN-1.0-2019-06-20 Raystation 9A Reference Manual. Stockholm, Sweden: Raysearch Laboratories AB (2019).

[14] Schneider U Pedroni E Lomax A. The Calibration of CT Hounsfield Units for Radiotherapy Treatment Planning. Phys Med Biol (1996) 41:111. doi: 10.1088/0031-9155/41/1/009 8685250

[15] Schneider W Bortfeld T Schlegel W. Correlation Between CT Numbers and Tissue Parameters Needed for Monte Carlo Simulations of Clinical Dose Distributions. Phys Med Biol (2000) 45:459. doi: 10.1088/0031-9155/45/2/314 10701515

[16] Wong JW Sharpe MB Jaffray DA Kini VR Robertson JM Stromberg JS. The Use of Active Breathing Control (ABC) to Reduce Margin for Breathing Motion. Int J Radiat Oncol Biol Phys (1999) 44:911–9. doi: 10.1016/S0360-3016(99)00056-5 10386650

[17] Li H Zhang X Park P Liu W Chang J Liao Z. Robust Optimization in Intensity-Modulated Proton Therapy to Account for Anatomy Changes in Lung Cancer Patients. Radiother Oncol (2015) 114:367–72. doi: 10.1016/j.radonc.2015.01.017 PMC4400219 25708992

[18] Chang JY Li H Zhu XR Liao Z Zhao L Liu A. Clinical Implementation of Intensity Modulated Proton Therapy for Thoracic Malignancies. Int J Radiat Oncol Biol Phys (2014) 90:809–18. doi: 10.1016/j.ijrobp.2014.07.045 PMC4252731 25260491

[19] Yang Z Chang Y Brock KK Cazoulat G Koay EJ Koong AC. Effect of Setup and Inter-Fraction Anatomical Changes on the Accumulated Dose in CT-Guided Breath-Hold Intensity Modulated Proton Therapy of Liver Malignancies. Radiother Oncol (2019) 134:101–9. doi: 10.1016/j.radonc.2019.01.028 PMC12131267 31005203

[20] Yang Z Zhang X Wang X Zhu XR Gunn B Frank SJ. Multiple-CT Optimization: An Adaptive Optimization Method to Account for Anatomical Changes in Intensity-Modulated Proton Therapy for Head and Neck Cancers. Radiother Oncol (2020) 142:124–32. doi: 10.1016/j.radonc.2019.09.010 PMC8564505 31564553

[21] Wang X Li H Zhu XR Hou Q Liao L Jiang B. Multiple-CT Optimization of Intensity-Modulated Proton Therapy–Is It Possible to Eliminate Adaptive Planning? Radiother Oncol (2018) 128:167–73. doi: 10.1016/j.radonc.2017.09.032 29054378

[22] Thummerer A Zaffino P Meijers A Marmitt GG Seco J Steenbakkers RJ. Comparison of CBCT Based Synthetic CT Methods Suitable for Proton Dose Calculations in Adaptive Proton Therapy. Phys Med Biol (2020) 65:095002. doi: 10.1088/1361-6560/ab7d54 32143207

[23] Kurz C Dedes G Resch A Reiner M Ganswindt U Nijhuis R. Comparing Cone-Beam CT Intensity Correction Methods for Dose Recalculation in Adaptive Intensity-Modulated Photon and Proton Therapy for Head and Neck Cancer. Acta Oncol (2015) 54:1651–7. doi: 10.3109/0284186X.2015.1061206 26198654

[24] Arai K Kadoya N Kato T Endo H Komori S Abe Y. Feasibility of CBCT-Based Proton Dose Calculation Using a Histogram-Matching Algorithm in Proton Beam Therapy. Physica Med (2017) 33:68–76. doi: 10.1016/j.ejmp.2016.12.006 27998666

[25] Lalonde A Winey B Verburg J Paganetti H Sharp GC. Evaluation of CBCT Scatter Correction Using Deep Convolutional Neural Networks for Head and Neck Adaptive Proton Therapy. Phys Med Biol (2020) 65:245022. doi: 10.1088/1361-6560/ab9fcb PMC8920050 32580174

[26] Neppl S Kurz C Köpl D Yohannes I Schneider M Bondesson D. Measurement-Based Range Evaluation for Quality Assurance of CBCT-Based Dose Calculations in Adaptive Proton Therapy. Med Phys (2021). doi: 10.1002/mp.14995 34032301

[27] De Ornelas M Xu Y Padgett K Schmidt RM Butkus M Diwanji T. CBCT-Based Adaptive Assessment Workflow for Intensity Modulated Proton Therapy for Head and Neck Cancer. Int J Particle Ther (2021) 7:29–41. doi: 10.14338/IJPT-D-20-00056.1 PMC8019579 33829071

[28] Spadea MF Pileggi G Zaffino P Salome P Catana C Izquierdo-Garcia D. Deep Convolution Neural Network (DCNN) Multiplane Approach to Synthetic CT Generation From MR Images—Application in Brain Proton Therapy. Int J Radiat Oncol Biol Phys (2019) 105:495–503. doi: 10.1016/j.ijrobp.2019.06.2535 31271823

